# Angiotensin-converting enzyme inhibitors and angiotensin II receptor blockers: potential allies in the COVID-19 pandemic instead of a threat?

**DOI:** 10.1042/CS20210182

**Published:** 2021-04-21

**Authors:** Fedor Simko, Tomas Baka

**Affiliations:** 1 Institute of Pathophysiology, Faculty of Medicine, Comenius University, Bratislava 81108, Slovak Republic; 2 Third Department of Internal Medicine, Faculty of Medicine, Comenius University, Bratislava 83305, Slovak Republic; 3 Institute of Experimental Endocrinology, Biomedical Research Center, Slovak Academy of Sciences, Bratislava 84505, Slovak Republic

**Keywords:** ACE2, ADAM17, angiotensin II type 1 receptor blocker, angiotensin-converting enzyme inhibitor, COVID-19

## Abstract

Angiotensin-converting enzyme 2 (ACE2) is the leading player of the protective renin–angiotensin system (RAS) pathway but also the entry receptor for severe acute respiratory syndrome coronavirus 2 (SARS-CoV-2). RAS inhibitors seemed to interfere with the ACE2 receptor, and their safety was addressed in COVID-19 patients. Pedrosa et al. (*Clin. Sci.* (*Lond.*) (2021), **135**, 465–481) showed in rats that captopril and candesartan up-regulated ACE2 expression and the protective RAS pathway in lung tissue. In culture of pneumocytes, the captopril/candesartan-induced ACE2 up-regulation was associated with inhibition of ADAM17 activity, counterbalancing increased ACE2 expression, which was associated with reduced SARS-CoV-2 spike protein entry. If confirmed in humans, these results could become the pathophysiological background for justifying RAS inhibitors as cornerstone cardiovascular protectives even during COVID-19 pandemic.

## Introduction

The renin–angiotensin–aldosterone system (RAAS) is a principal player in the acute stress reaction. However, if RAAS is inappropriately activated, it can induce cellular hypertrophy and fibrosis, resulting in pathologic remodelling of target organs with cardiovascular prognosis deterioration [1,2]. In line with the abovementioned, the effort of experimental and clinical cardiology has been focused on curbing RAAS overactivity to a desirable level. First peroral angiotensin-converting enzyme (ACE) inhibitor (ACEI) captopril was discovered in 1972 [3] and later the angiotensin II (Ang II) type 1 (AT1) receptor blocker (ARB) was disclosed [4]. ACEI and ARB mitigate Ang II formation or effect. The discovery of ACEI/ARB has brought about a revolution in the treatment of hypertension, hypertensive and failing heart, hypertensive and diabetic nephropathy and in the prevention of atherosclerotic complications in recent decades. Besides the obvious haemodynamic and antiproliferative actions, a number of pleiotropic effects in terms of blunting coagulation, oxidative stress or inflammation play a significant role in the versatile benefits of ACEI/ARB [1,2].

## SARS-CoV-2, Ang II, Ang 1-7 interaction

The outbreak of the COVID-19 pandemic has not only afflicted everyday life at manifold levels but also introduced novel therapeutic challenges [5,6]. Unfortunately, a number of clinical trials testing antiviral, anti-inflammatory and immunomodulatory substances have brought more disappointment than delight. Unexpectedly, ACEI/ARB evoked worldwide concerns regarding the safety and justification of their usage during the COVID-19 pandemic. Since the 1990s, evidence has been emerging that the potentially deleterious action of the classical ACE/Ang II/AT1R pathway is being opposed by the alternative ACE2/Ang1-7/MasR route counterbalancing the Ang II-mediated haemodynamic burden, remodelling and inflammation by promoting natriuresis and vasodilation and inhibiting proliferation and immune reaction. ACE2 is a link between the RAAS and severe acute respiratory syndrome coronavirus 2 (SARS-CoV-2) infection with a Janus face [7]. On the one hand, ACE2 transforms Ang II to Ang 1-7 with an anti-inflammatory nature. On the other hand, ACE2 is a binding receptor and entry route for SARS-CoV-2 [6,8,9]. Since the findings of experimental studies indicated that ACEI/ARB increased cellular membrane-bound ACE2 (mACE2) expression, opinions have emerged about whether ACEI/ARB may heighten susceptibility to SARS-CoV-2 infection and worsen its prognosis; thus, the justification of renin–angiotensin system (RAS) inhibition in COVID-19 patients was addressed [10]. Considering the other side of the coin, ACEI/ARB might potentially be of benefit. They reduce the deleterious effects of Ang II and support ACE2-mediated conversion of Ang II into Ang 1-7 or Ang I to Ang 1-9; both alternative angiotensins attenuate inflammation and proliferation via Mas or AT2 receptor stimulation [6,11]. Furthermore, splitting thymosin β4 with prolyl oligopeptidase gives rise to N-acetyl-seryl-aspartyl-lysyl-proline (AcSDKP), which exerts a beneficial effect on tissue structure by direct actions curbing inflammation and excessive fibrosis in the heart, vessels and kidney. AcSDKP is an alternative substrate for ACE; thus, ACEI reduce degradation and hence increase the availability of AcSDKP, which supposedly participates in the protection by ACEI [12]. Although experimental findings supporting or refusing the harm or benefit of ACEI/ARB treatment in COVID-19 were rare and clinical studies were lacking, several considerations endorsed the continuation of ACEI/ARB therapy. Most importantly, large clinical trials have provided sufficient evidence that patients with cardiovascular pathologies, hypertension or diabetes benefit from RAAS inhibition. Moreover, the withdrawal of these drugs may induce a rebound phenomenon resulting in haemodynamic or atherosclerotic plaque instability. Additionally, in patients with cardiovascular pathologies and concomitant neurohumoral activation, there is virtually no adequate replacement therapy, especially in the severely ill population with saturated therapeutic schedule [13,14]. Thus, international societies of cardiology recommended maintaining the ACEI/ARB treatment of cardiovascular pathologies in patients with COVID-19 [14].

## No double-edged sword effect with ACEI or ARB

**Figure 1:**
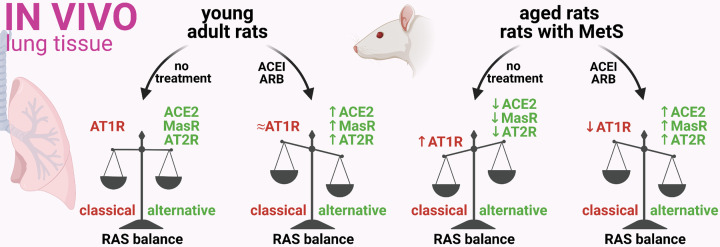
ACEI/ARB treatment up-regulated the protective RAS axis in the lungs of young rats and also in aged rats or rats with MetS Three weeks of captopril (ACEI) or candesartan (ARB) treatment shifted the RAS balance towards the alternative axis in the lungs of young rats, as well as aged rats and rats with MetS in terms of up-regulating the expression of ACE2, MasR and AT2R [15]. Created with BioRender.com.

A recently published pioneer experimental study by Pedrosa et al. [15] provides the first mechanistic insight into RAS modulation by ACEI or ARB in COVID-19 and its co-morbidities. The study showed *in vivo* that three weeks of captopril (ACEI) or candesartan (ARB) treatment up-regulates the ACE2 expression associated with increased ACE2 enzymatic activity in the lungs of young healthy rats. In addition, both drugs up-regulated the level of MasR, and candesartan also increased the expression of AT2R, with both receptors exerting potential protection. In untreated aged rats and rats with metabolic syndrome (MetS), however, the RAS balance in the lung tissue was shifted towards the pro-inflammatory axis: decreased expression of ACE2 and an increased level of AT1R in both animal models, and also decreased expression of MasR and AT2R in aged rats. Captopril or candesartan reversed RAS dysregulation in the lungs of aged rats and rats with MetS by enhancing ACE2 expression and decreasing AT1R expression to levels similar to those found in young adult rats, while also increasing the expression of both MasR and AT2R over the levels found in their young adult counterparts (Figure 1).

**Figure 2:**
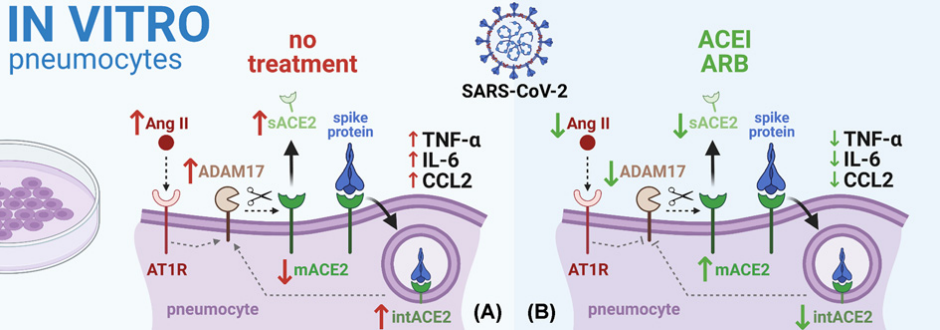
In culture of pneumocytes, the ACEI/ARB-induced mACE2 up-regulation was associated with ADAM17 inhibition and reduced SARS-CoV-2 spike protein entry (**A**) In *in vitro* experiment, binding the SARS-CoV-2 spike protein on mACE2 resulted in spike protein internalization associated with the reduction in mACE2 levels, whereas the levels of short ACE2 in pneumocytes (intACE2) and sACE2 in culture medium increased. Furthermore, spike protein induced an increase in ADAM17 enzymatic activity and pro-inflammatory cytokine levels (TNF-α, IL-6 and CCL2) in culture medium. (**B**) ACEI/ARB prevented spike protein internalization and the decline of mACE2 levels, suggesting reduced mACE2 shedding. Importantly, ACEI/ARB hampered the spike protein-induced increase in ADAM17 activity. Moreover, ACEI/ARB alleviated the spike protein-induced pro-inflammatory cytokine response [15]. Abbreviation: intACE2, internalized cellular ACE2. Created with BioRender.com.

*In vitro*, captopril and candesartan increased the expression of ACE2 in human type-II pneumocytes, thus corroborating the results from *in vivo* experiments. After binding the SARS-CoV-2 spike protein on ACE2, the spike protein internalization associated with reduced mACE2 levels and enzymatic activity ensued, and the levels of short ACE2 in pneumocytes and soluble ACE2 (sACE2) in culture medium increased. Pre-treatment with captopril or candesartan prevented spike protein internalization and normalized mACE2 levels and enzymatic activity, suggesting reduced mACE2 shedding. Indeed, treatment with spike protein increased ADAM17 enzymatic activity in the culture medium, thus facilitating mACE2 shedding; captopril and candesartan hampered the effect of the spike protein on ADAM17 activity, thus curbing mACE2 shedding. Furthermore, treatment with the spike protein was coupled with increased levels of pro-inflammatory cytokines TNF-α, IL-6 and CCL2 in culture medium, which were reduced by captopril and candesartan (Figure 2).

Taken together, the results of the study by Pedrosa et al. [15] support the presumption about SARS-CoV-2-induced RAS dysregulation and refute the hypothesis on adverse effects of RAS modulation by ACEI or ARB in COVID-19. In fact, correcting the SARS-CoV-2 spike protein-induced RAS imbalance by captopril or candesartan was shown to be potentially protective by decreasing the viral entry despite the increased ACE2 expression and alleviating pro-inflammatory cytokine release. Modulation of ADAM17 activity appears to play a determining role in the protective mechanism by ACEI or ARB. ADAM17 is a key sheddase that cleaves mACE2 and participates in viral entry mechanisms and pro-inflammatory cytokine release [16]. Indeed, the SARS-CoV-2-associated RAS imbalance might also increase ADAM17 activity via the Ang II/AT1R/Nox pathway [17] leading to additional proteolytic cleavage of mACE2 and potentially triggering a positive feedback pathway interconnecting mACE2 shedding–Ang II–ADAM17 activation [18]. On the contrary, ACEI and ARB, besides switching the RAS balance towards the anti-inflammatory alternative axis, act as ADAM17 inhibitors, thus protecting the mACE2 from being shed, hindering viral entry and contributing to cytokine response alleviation. Interestingly, dedicated ADAM17 inhibitors exerted similar beneficial effects in SARS-CoV infection and were proposed as a candidate for antiviral therapy [19].

The data are partly in agreement with previous findings in patients with heart failure (HF). In two independent cohorts of HF patients, ACEI or ARB did not increase plasma levels of ACE2 [20]. Although the relationship to COVID-19 was not investigated, these clinical findings add another piece into the puzzle on the safety of ACEI/ARB.

Some suggestions for future research are emerging: Could the effects presented by Pedrosa et al. also be expected with mineralocorticoid receptor antagonists, such as spironolactone? Is the protection of mACE2 associated with adequate inflammatory response? As sACE2 maintains its catalytic activity [21], while representing a decoy for SARS-CoV [22], is the reduced sACE2 plasma level indicative of a clinical benefit in COVID-19?

## Future directions of ACEI/ARB in relation to COVID-19

The elegant paper by Pedrosa et al. [15], with its encouraging data on RAS inhibition, represents an experimental platform for continuing the ACEI/ARB treatment of cardiovascular pathologies during COVID-19. Indeed, a number of currently available epidemiological studies and meta-analyses are revealing that ACEI/ARB treatment of cardiovascular disorders in COVID-19 patients is not associated with increased infection rate and disease severity [23]. A large and recent international, open science cohort analysis, based on electronic health records from Spain and U.S.A., involved 1,355,349 hypertensive patients aged 18 years or older with a prescription of ACEI or ARB (target cohort) or calcium channel blockers and thiazid diuretics (comparator cohort). No significant increased risk of COVID-19 diagnosis or hospitalization-related outcomes associated with the use of ACEI/ARB was observed [24]. A number of randomized controlled trials are currently underway to improve clinical decision making for the use of ACEI/ARB in COVID-19 patients. Since some studies may be underpowered, the International Society of Hypertension elaborated a prospective meta-analysis protocol for randomized trials with RAS inhibitors in COVID-19 patients to pool and analyze data from the current randomized controlled trials [23].

The use of ACEI/ARB in COVID-19 seems to be safe, and one might expect even some benefit in SARS-CoV-2 infected patients. Yet, predictions should be made with great care. The difficulty in assuming the results of the ongoing trials is related to the complex nature of the RAAS, as SARS-CoV-2 or a therapeutic intervention aimed at the RAAS can influence several protective or deleterious pathways. In addition, the shortage of experimental data aggravates the interpretation and one should consider several pros and cons coming with RAAS inhibition:

Bradykinin is a substrate of both ACE and ACE2 [25,26]. ACEI reduce bradykinin splitting by ACE, thus increasing its bioavailability. Bradykinin’s pro-inflammatory action can increase the side effects of ACEI [25] and counterbalance the benefit of the anti-inflammatory action of Ang II down-regulation. SARS-CoV-2-induced inhibition of ACE2 may contribute to an excess of bradykinin, thus stimulating B1 and B2 receptors and mediating pro-inflammatory action. Although the inhibition of the bradykinin pathway by icatibant or C1 esterase/kallikrein inhibitor failed to reduce the time to clinical improvement, both compounds were safe and showed improvement of lung computed tomography scores suggesting improved disease recovery [26].

Hope is endorsed by a piece of evidence indicating a potential benefit of ACEI/ARB in inflammatory lung diseases. A large, retrospective cohort study of 215,225 patients with chronic obstructive pulmonary disease indicated that treatment with ACEI or ARB reduced pulmonary infections and structural damage [27]. A population-based cohort study involving 254,485 elderly patients found that, compared with other antihypertensive drugs, the newly prescribed antihypertensive treatment with ACEI/ARB decreased the risk of hospitalization with pneumonia within 90 days following treatment [28].

Although aldosterone is an integral part of the RAAS, little attention is devoted to its modulation in COVID-19. Spironolactone, a mineralocorticoid receptor antagonist, might attenuate the viral entry into cells. Before binding to mACE2, the virus spike protein should be processed by transmembrane protease receptor serine type 2 (TMPRSS2), furine or plasmin, which are down-regulated by spironolactone [29]. Furthermore, the antifibrotic and anti-inflammatory effect of spironolactone could attenuate pulmonary and myocardial fibrosis in the restitution phase of COVID-19.

Curbing inflammatory processes may be of an ambiguous nature. In the initial period, an Ang II peak may be desirable for fighting the spread of infection via inflammation. In the later course, the dominance of the Ang1-7/MasR pathway could calm down the immune reaction and support reparative processes [30]. Several strategies to preserve the RAAS balance may be of value. Importantly, using AT2 agonists could be a rational strategy to oppose the pro-inflammatory action of Ang II in COVID-19 patients [6]. Treatment with soluble recombinant ACE2 may trap the virus, thus curbing the spread of the infection, and limiting pulmonary tissue inflammation [22]. Furthermore, SARS-CoV-2-binding to mACE2 in alveolar cells may be competitively inhibited by using small extracellular vesicles processed to bind the virus [31].

The results of ongoing experimental and clinical studies will shed more light on the matter, whether the therapeutic interventions focused on the RAAS can gain an important position in the fight against SARS-CoV-2 infection.

## Abbreviations

ACE2: angiotensin-converting enzyme 2
ACEI: angiotensin-converting enzyme inhibitor
ADAM17: A disintegrin and metalloprotease 17
Ang: angiotensin
ARB: angiotensin II receptor blocker
AT1R: angiotensin II type 1 receptor
AT2R: angiotensin II type 2 receptor
COVID-19: Coronavirus disease 2019
IL-6: interleukin 6
mACE2: membrane-bound ACE2
MasR: Mas receptor
MetS: metabolic syndrome
RAAS: renin–angiotensin–aldosterone system
RAS: renin–angiotensin system
sACE2: soluble ACE2
SARS-CoV-2: severe acute respiratory syndrome coronavirus 2
